# Large-scale sporting events during the COVID-19 pandemic: insights from the FIFA World Cup 2022 in Qatar with an analysis of patterns of COVID-19 metrics

**DOI:** 10.5114/biolsport.2023.131109

**Published:** 2023-09-07

**Authors:** Ismail Dergaa, Helmi Ben Saad, Piotr Zmijewski, Ramdan Abdelmoez Farhat, Mohamed Romdhani, Amine Souissi, Jad Adrian Washif, Morteza Taheri, Noomen Guelmami, Nizar Souissi, Karim Chamari, Samya Ahmad Al Abdulla

**Affiliations:** 1 Primary Health Care Corporation (PHCC), Doha, Qatar; 2 Research Unit Physical Activity, Sport, And Health, UR18JS01, National Observatory of Sport, Tunis 1003, Tunisia; 3 High Institute of Sport and Physical Education, University of Sfax, Sfax, Tunisia; 4 University of Sousse, Farhat HACHED Hospital, Faculty of Medicine of Sousse, Research Laboratory (LR12SP09) “Heart Failure” Sousse, Tunisia; 5 Jozef Pilsudski University of Physical Education in Warsaw, Warsaw, Poland; 6 Faculty of Medicine, Kafrelsheikh University, Kafrelsheikh, 33511, Egypt; 7 Motricité-Interactions-Performance, MIP, UR4334, Le Mans University, Le Mans, France; 8 Sports Performance Division, Institut Sukan Negara Malaysia (National Sports Institute of Malaysia), Kuala Lumpur, Malaysia; 9 Department of Motor Behavior, Faculty Of Sport Sciences And Health, University of Tehran, Tehran, Iran; 10 Postgraduate School of Public Health, Department of Health Sciences (DISSAL), University of Genoa, Genoa, Italy; 11 Department of Human and Social Sciences, High Institute of Sport and Physical Education of Kef, University of Jendouba, Kef 7100, Tunisia; 12 Aspetar, Orthopedic and Sports Medicine Hospital, FIFA Medical Centre of Excellence, Doha, Qatar

**Keywords:** Epidemiological analysis, Herd immunity, Infectious disease transmission, Mass gathering, Pandemic management, Soccer, SARS-CoV-2

## Abstract

The 2022 FIFA World Cup (FIFA-WC) held in Qatar presented unique challenges, given the potential for rapid transmission of coronavirus disease 2019 (COVID-19) among over 1.4 million international fans attending the event. This study aimed to investigate the impact of the FIFA-WC 2022 on COVID-19 cases, deaths, and reproduction rate (R0) in Qatar. Additionally, it sought to understand the implications of hosting large-scale events during a pandemic without COVID-19 restrictive measures, providing critical insights for future decision-making. Data from “Our World in Data” were analysed for three distinct periods: one week before the FIFA-WC (week-preWC), the four weeks of the event (week-1WC to week-4WC), and one week after (week-postWC). The results revealed a significant increase in COVID-19 cases during week-3WC and week-4WC (compared to week-preWC) in Qatar, followed by a subsequent decrease during the week-postWC. Notably, Qatar experienced a more pronounced surge in positive cases than the global trend. Regarding COVID-19-related deaths, Qatar’s peak occurred during week-2WC, while globally deaths peaked from week-3WC to week-postWC. Nevertheless, Qatar’s death toll remained relatively low compared to the global trend throughout the event. The findings highlight that the FIFA-WC 2022 in Qatar demonstrated the feasibility of organizing large-scale sporting events during a pandemic with appropriate measures in place. They emphasize the importance of high vaccination coverage, continuous monitoring, and effective collaboration between event organizers, healthcare authorities, and governments. As such, the event serves as a valuable model for future gatherings, underlining the significance of evidence-based decision-making and comprehensive public health preparedness.

## INTRODUCTION

The outbreak of coronavirus disease 2019 (COVID-19) has had a significant impact on many aspects of society, including sports events [1–3]. As COVID-19 rapidly spread, many significant competitions, including the Olympic Games and the UEFA Champions League, were either cancelled or postponed [4, 5]. The effects of the pandemic on sports events have given rise to several concerns, such as the financial losses suffered by organizers, interruption of athletes’ training and competition routines, and the negative impact on their mental and physical health [3, 5, 6].

The FIFA World Cup (FIFA-WC) is the largest international sports event, bringing together a diverse range of attendees from all over the world. Due to its scale and scope, hosting this event presents unique challenges to organizing countries in normal times, let alone in times of sanitary threat [4, 5]. Indeed, the 22^nd^ FIFA-WC 2022, which took place in Qatar from 20 November to 18 December 2022, was unique in several ways. Firstly, this event was the first FIFA-WC to ever be hosted during an ongoing pandemic (i.e. COVID-19) [4, 5]. This was one of the key challenges because, due to close human proximity, there was a potential for rapid spread of highly transmissible infectious disease from imported or endemic communicable diseases. Secondly, Qatar FIFA-WC 2022 was the first World Cup to be held in such a small nation in terms of population size and geographic area [2, 5]. Besides the challenges that have been faced previously, it is worth noting that, unlike most major sporting events previously held during the pandemic, the FIFA-WC took place without any sanitary restrictions, as Qatar lifted all travel and local restrictions related to COVID-19 in September 2022 [7].

Therefore, the aims of this study were to: i) investigate the impact of organizing the FIFA-WC 2022 on COVID-19 cases, deaths, and reproduction rate (R0) in Qatar, and ii) understand the implications of hosting large-scale events during a pandemic in the absence of COVID-19 restrictive measures, for critical decision-making in the future.

## MATERIALS AND METHODS

### Event

The 22^nd^ edition of FIFA-WC was held in Qatar from November 20^th^ to December 18^th^, 2022. The event was scheduled for November and December to avoid Qatar’s summer extreme heat. The tournament featured 64 matches played in eight venues in Doha and the surrounding areas of the capital over 29 days. The average turnout per match was 53,191 spectators, with a total attendance of 3,404,252 fans [8]. In addition, over 1.8 million fans watched the live broadcast of games and festivities at the FIFA Fan Festival held in Al Bidda Park, Doha [9]. The final match at Lusail Stadium drew an attendance of 88,966 spectators, and the FIFA-WC was viewed by over 5 billion TV viewers globally.

### Data sources

We acquired data from publicly available sources, including government websites such as the Ministry of Public Health (MOPH) in Qatar, which provided local COVID-19 data for the country [10]. We also used other academic data sets, such as ‘’Our World in Data’’, to investigate COVID-19 data in other countries and obtain the daily number of international newly infected cases, deaths, and the R0 [11]. Our primary dataset was ‘’Our World in Data’’, which we cross-checked with the data provided by the MOPH to ensure accuracy. R0 data were not available on the MOPH website; therefore we retrieved the data from the ‘’Our World in Data’’ website. In addition, we searched PubMed Central (PMC, last accessed on the 7^th^ April 2023) for peer-reviewed studies on the impact of mass gatherings on the spread of COVID-19, which we used to write up our manuscript.

### Data collection methods

The aforementioned sources provided us with the necessary data, which we subsequently organized into an Excel spreadsheet for analysis. The data were collected during three distinct time periods: before, during, and after the FIFA-WC 2022. Specifically, we collected data one week before the FIFA-WC (i.e. week-preWC: November 13^th^ to 19^th^), during the FIFA-WC (i.e. four weeks from November 20^th^ to December 18^th^, totalling 29 days), and one week after the FIFA-WC (i.e. week-postWC: December 19^th^ to 25^th^). The period of “during FIFA-WC” was divided into four weeks (i.e. week-1WC: November 20^th^ to 26^th^; week-2WC: November 27^th^ to December 3^rd^; week-3WC: December 4^th^ to 10^th^; and week-4WC: December 11^th^ to 18^th^). This allowed us to compare COVID-19 cases, deaths, and R0 in Qatar to the world population before, during, and after the FIFA-WC event.

In addition to conducting a statistical analysis, we intentionally created a graph that features data – 10 days before the FIFA-WC (from November 10^th^ to 19^th^), during the 29 days of the FIFA-WC (from November 20^th^ to December 18^th^), and 10 days after the FIFA-WC (from December 19^th^ to 28^th^) – from multiple countries across four continents: Asia (Qatar, Saudi Arabia, United Arab Emirates (UAE) and Russia), Africa (Tunisia and South Africa), Europe (United Kingdom), America (Canada and Mexico). The latter countries represented the region and different continents, and with the exception of Russia, South Africa and UAE, had their national teams participating in the event. Our objective was to illustrate to readers the approximate patterns of COVID-19 worldwide in comparison to Qatar.

### Data

We analysed the following three key metrics related to COVID-19 in Qatar: daily new confirmed COVID-19 cases per million people, daily new confirmed COVID-19 related deaths per million people, and effective R0. R0 measures the average number of new infections caused by a single infected individual [12]. If R0 is > 1, the infection is able to spread in the population. If R0 ≤ 1, the number of cases occurring in the population will gradually decrease until it reaches zero [12].

### Ethical approval

Ethical approval was sought for this study by consulting with the Institutional Review Board (IRB) of the Primary Health Care Corporation (PHCC) on January 30^th^, 2023. As our study only involved the utilization of publicly available data, the IRB granted us approval of exempted review process application.

### Statistical analysis

The daily COVID-19 related new confirmed cases and deaths, and effective R0 for the world and Qatar during the six weeks were expressed as mean ± standard deviation (95% confidence interval). Comparisons between the six weeks were made via an analysis of variance (ANOVA). If p was < 0.05, a post-hoc analysis (Tukey HSD test) was performed. All analyses were carried out using STATISTICA software version 12. The significance level was set at 5% (p < 0.05).

## RESULTS

### Confirmed cases, COVID-19 related confirmed deaths, and effective R0 in Qatar, the world, and selected countries

**FIG. 1:**
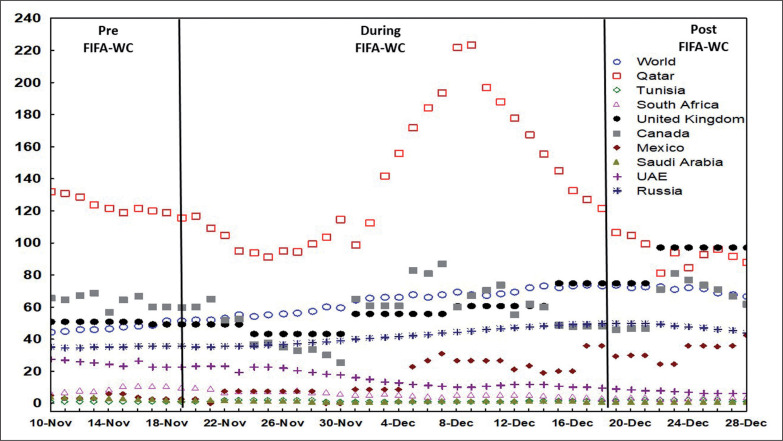
Evolution of the confirmed COVID-19 related cases per million people in Qatar, the world, and multiple countries 10 days pre the FIFA World-Cup (WC), during the FIFA-WC, and 10 days post the FIFA-WC. COVID-19: Coronavirus Disease of 2019

**FIG. 2:**
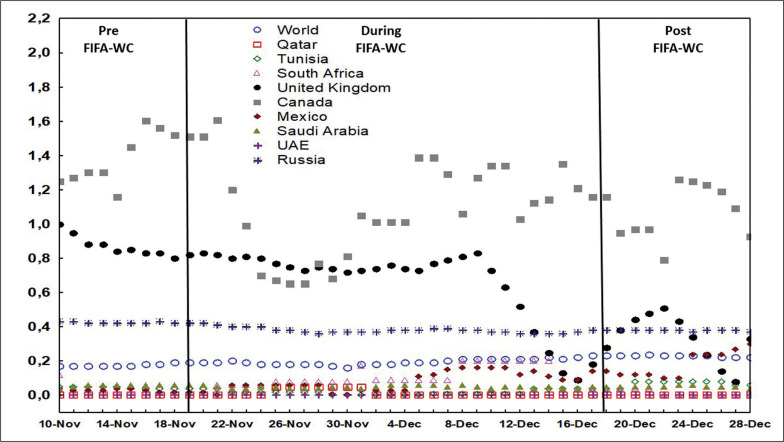
Evolution of the COVID-19 related confirmed deaths per million people in Qatar, the world, and multiple countries 10 days pre the FIFA World-Cup (WC), during the FIFA-WC, and 10 days post the FIFA-WC. COVID-19: Coronavirus Disease of 2019.

**FIG. 3:**
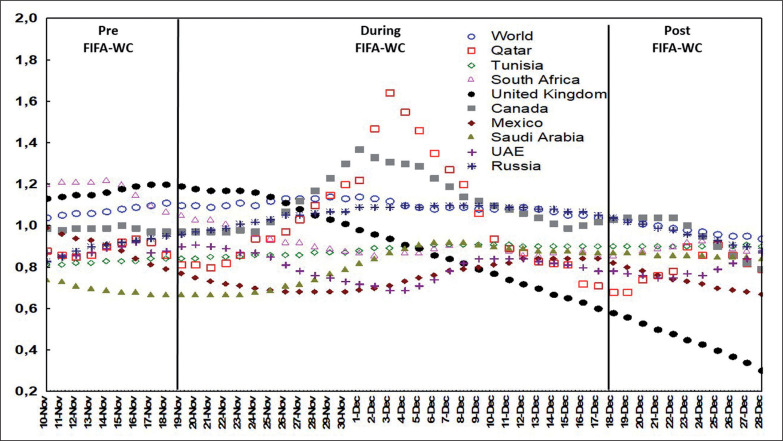
Evolution of effective COVID-19 reproduction rate (R0) in Qatar, the world, and multiple countries 10 days pre the FIFA World-Cup (WC), during the FIFA-WC, and 10 days post the FIFA-WC. COVID-19: Coronavirus Disease of 2019.

Figures 1, 2, and 3 illustrate the evolution of the confirmed COVID-19 cases per million people (Figure 1), confirmed deaths per million people (Figure 2), and the effective R0 (Figure 3) in Qatar, the world, and selected countries 10 days before the FIFA-WC, during the FIFA-WC, and 10 days after the FIFA-WC.

Table 1 presents the COVID-19 related confirmed cases per million people, confirmed deaths per million people, and effective R0 before, during, and after the FIFA-WC 2022 in Qatar and the world.

**TABLE 1:** COVID-19 confirmed cases per million people, confirmed deaths per million people, and reproduction rate pre, during, and post the FIFA world-cup (WC) 2022: data from the world and Qatar.

Periods	Pre WC (7 days before)	During WC (29 days)				Post WC (7 days after)	ANOVA p-value	Tukey HSD test p-value
Weeks	W-preWC: November 13 to 19	W-1WC: November 20 to 26	W-2WC: November 27 to December 3	W-3WC: December 4 to 10	W-4WC: December 11 to 18	W-postWC: December 19 to 25		
**CONFIRMED CASES**								
**World**	48.70 ± 2.18 (46.68 to 50.72)	54.09 ± 1.52 (52.69 to 55.50)	61.60 ± 3.97 (57.92 to 65.28)	67.66 ± 1.24 (66.52 to 68.81)	72.14 ± 2.04 (70.43 to 73.84)	72.45 ± 0.85 (71.67 to 73.24)	F_(5, 37)_ = 142 p = 0.0001	**^abcdefghijklmn^**
**Qatar**	120.23 ± 2.56 (117.87 to 122.60)	101.03 ± 9.43 (92.30 to 109.75)	109.55 ± 16.10 (94.66 to 124.44)	192.86 ± 24.67 (170.05 to 215.67)	152.15 ± 24.40 (131.75 to 172.55)	95.05 ± 9.50 (86.27 to 103.84)	F_(5, 37)_ = 35 p = 0.0001	**^cdghjkmno^**
**CONFIRMED DEATHS**								
**World**	0.18 ± 0.01 (0.17 to 0.19)	0.19 ± 0.01 (0.18 to 0.19)	0.18 ± 0.01 (0.17 to 0.18)	0.20 ± 0.01 (0.19 to 0.21)	0.22 ± 0.01 (0.21 to 0.22)	0.23 ± 0.00 (0.23 to 0.23)	F_(5, 37)_ = 54 p = 0.0001	**^cdehijklmn0^**
**Qatar**	0.00 ± 0.00	0.01 ± 0.02 (-0.01 to 0.04)	0.04 ± 0.02 (0.01 to 0.06)	0.00 ± 0.00	0.00 ± 0.00	0.00 ± 0.00	F_(5, 37)_ = 8 p = 0.0001	**^bjkl^**
**REPRODUCTION RATE**								
**World**	1.09 ± 0.02 (1.07 to 1.10)	1.11 ± 0.01 (1.09 to 1.12)	1.13 ± 0.01 (1.13 to 1.14)	1.09 ± 0.01 (1.08 to 1.09)	1.06 ± 0.02 (1.04 to 1.08)	0.99 ± 0.02 (0.97 to 1.01)	F_(5, 37)_ = 58 p = 0.0001	**^behijkln^**
**Qatar**	0.89 ± 0.05 (0.84 to 0.93)	0.88 ± 0.07 (0.81 to 0.94)	1.26 ± 0.22 (1.06 to 1.46)	1.26 ± 0.22 (1.06 to 1.46)	0.79 ± 0.08 (0.73 to 0.86)	0.80 ± 0.09 (0.72 to 0.88)	F_(5, 37)_ = 18 p = 0.0001	**^bcfgklmn^**

**ANOVA**: Analysis of variance. **COVID-19:** Coronavirus disease of 2019. **W**: Week.

Data were mean ± standard deviation (95% confidence interval).

ANOVA: Comparison between the 6 weeks for Qatar and the world.

Tukey HSD test (p < 0.005):

^a^ W-preWC vs. W-1WC; ^b^. W-preWC vs. W-2WC; ^c^ W-preWC vs. W-3WC; ^d^ W-preWC vs. W-4WC; ^e^ W-preWC vs. W-postWC;

^f^ W-1WC vs. W-2WC; ^g^ W-1WC vs. W-3WC; ^h^ W-1WC vs. W-4WC; ^i^ W-1WC vs. W-postWC;

^j^ W-2WC vs. W-3WC; ^k^ W-2WC vs. W-4WC; ^l^ W-2WC vs. W-postWC;

^m^ W-3WC vs. W-4WC; ^n^ W-3WC vs. W-postWC;

^o^ W-4WC vs. W-postWC

**FIG. 4:**
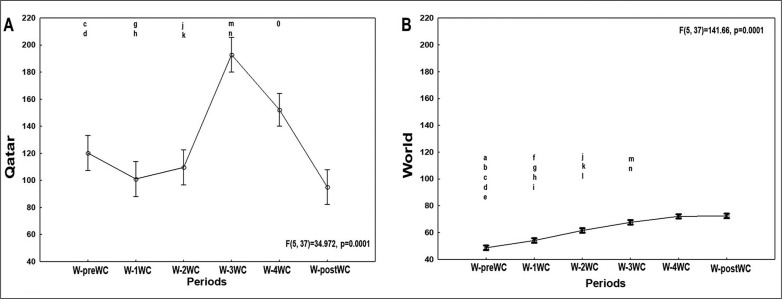
Evolution of the COVID-19 confirmed cases per million people in Qatar (Figure 4A), and in the world (Figure 4B), one-week pre the FIFA world-cup (W-preWC), four weeks during the FIFA-WC (W-1WC to W-4WC), and one week post the FIFA-WC (W-postWC). Data were mean ± standard deviation (95% confidence interval). COVID-19: Coronavirus Disease of 2019.W: week. Analysis of variance (ANOVA): comparison between the 6 weeks for each region. Tukey HSD test (p < 0.005): W-preWC vs. ^a^ W-1WC; ^b^ W-2WC; ^c^ W-3WC; ^d^ W-4WC; ^e^ W-postWC; W-1WC vs. ^f^ W-2WC; ^g^ W-3WC; ^h^ W-4WC; ^i^ W-postWC; W-2WC vs. ^j^ W-3WC; ^k^ W-4WC; ^l^ W-postWC; W-3WC vs. ^m^ W-4WC; ^n^ W-postWC; ^o^ W-4WC vs. W-postWC.

**FIG. 5:**
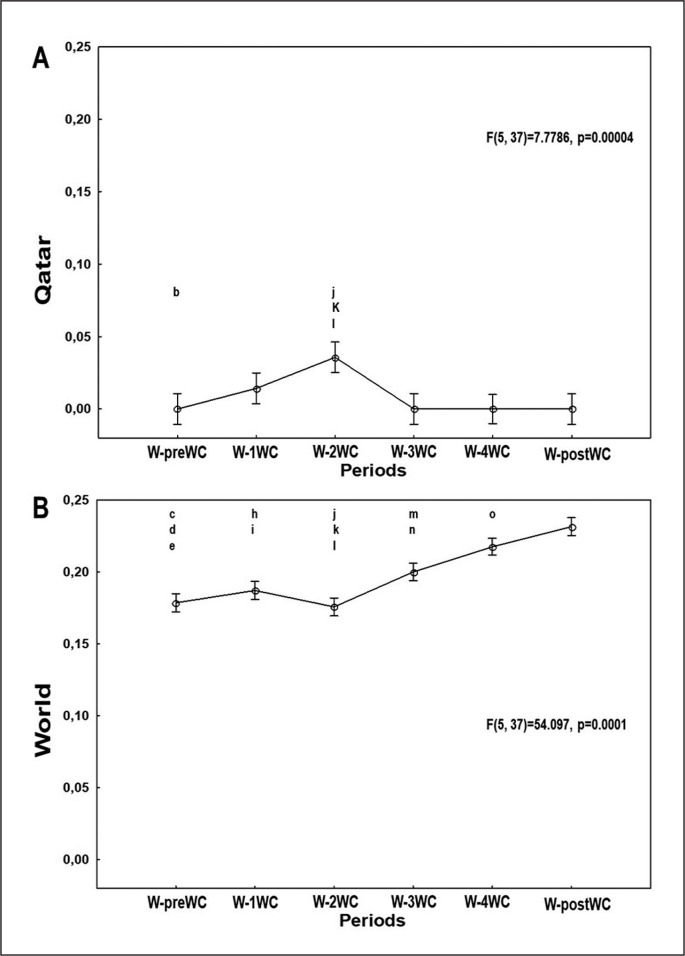
Evolution of the COVID-19 confirmed deaths per million people in Qatar (Figure 5A), and in the world (Figure 5B), one-week pre the FIFA World-Cup (W-preWC), four weeks during the FIFA-WC (W-1WC to W-4WC), and one week post the FIFA-WC (W-postWC). Data were mean ± standard deviation (95% confidence interval). COVID-19: Coronavirus Disease of 2019.W: week. “Analysis of variance (ANOVA): Tukey HSD test (p < 0.005): W-preWC vs. ^a^ W-1WC; ^b^ W-2WC; ^c^ W-3WC; ^d^ W-4WC; ^e^ W-postWC; W-1WC vs. ^f^ W-2WC; ^g^ W-3WC; ^h^ W-4WC; ^i^ W-postWC; W-2WC vs. ^j^ W-3WC; ^k^ W-4WC; ^l^ W-postWC; W-3WC vs. ^m^ W-4WC; ^n^ W-postWC; ^o^ W-4WC vs. W-postWC.

**FIG. 6:**
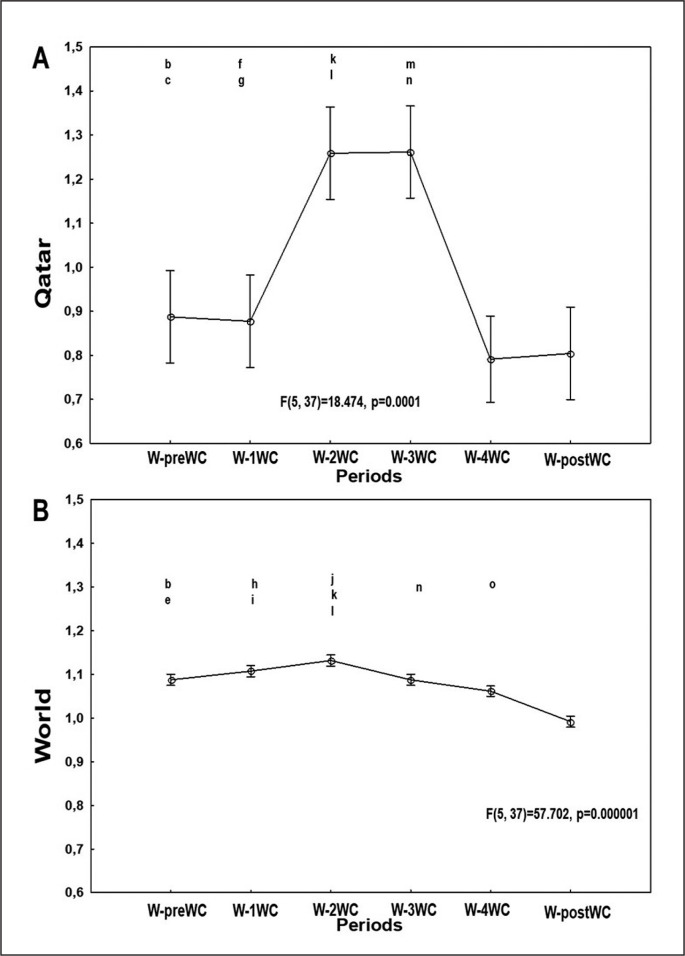
Evolution of the COVID-19 reproduction rate in Qatar (Figure 6A), and in the world (Figure 6B), one-week pre the FIFA World-Cup (W-preWC), four weeks during the FIFA-WC (W-1WC to W-4WC), and one week post the FIFA-WC (W-postWC). Data were mean ± standard deviation (95% confidence interval). COVID-19: Coronavirus Disease of 2019. W: week. Analysis of variance (ANOVA): Tukey HSD test (p < 0.005): W-preWC vs. ^a^ W-1WC; ^b^ W-2WC; ^c^ W-3WC; ^d^ W-4WC; ^e^ W-postWC; W-1WC vs. ^f^ W-2WC; ^g^ W-3WC; ^h^ W-4WC; ^i^ W-postWC; W-2WC vs. ^j^ W-3WC; ^k^ W-4WC; ^l^ W-postWC; W-3WC vs. ^m^ W-4WC; ^n^ W-postWC; ^o^ W-4WC vs. W-postWC

Figures 4, 5, and 6 present the evolution of the confirmed cases per million people (Figure 4), confirmed deaths per million people (Figure 5), and the effective R0 (Figure 6) in Qatar and the world one week before the FIFA-WC, four weeks during the FIFA-WC, and one week after the FIFA-WC.

### Confirmed cases in Qatar and in the world

ANOVA analysis revealed significant differences between the six weeks in both Qatar and the world (Table 1, Figure 4).

In Qatar (Table 1, Figure 4A), the mean number of confirmed cases increased significantly during week-3WC and week-4WC compared to : i) week-preWC; ii) week-1WC; and iii) week-2WC. The mean number of confirmed cases decreased significantly during : i) week-4WC and week-postWC compared to week-3WC; and ii) week-postWC compared to week-4WC.

In the world (Table 1, Figure 4B), the mean number of confirmed cases increased significantly during : i) week-1WC to week-4WC and week-postWC compared to week-preWC; ii) week-2WC to week-4WC and week-postWC compared to week-1WC; iii) week-3WC and week-4WC and week-postWC compared to week-2WC; and iv) week-4WC and week-postWC compared to week-3WC. The mean number of confirmed cases was comparable during week-4WC and week-postWC.

### Confirmed deaths in Qatar and in the world

ANOVA revealed significant differences between the six weeks in both Qatar and he world (Table 1, Figure 5).

In Qatar (Table 1, Figure 5A), the mean number of confirmed deaths was significantly higher during week-2WC compared to week-preWC, week-3WC, week-4WC, and week-postWC.

In the world (Table 1, Figure 5B), the mean number of confirmed deaths increased significantly during: i) week-3WC, week-4WC, and week-postWC compared to week-preWC; ii) week-4WC and week-postWC compared to week-1WC; iii) week-3WC, week-4WC, and week-postWC compared to week-2WC; iv) week-4WC and week-postWC compared to week-3WC; and v) week-postWC compared to week-4WC.

### Effective R0 in Qatar and in the world

ANOVA analysis revealed significant differences between the six weeks in both Qatar and the world (Table 1, Figure 6).

In Qatar (Table 1, Figure 6A), the mean value of effective R0 i) increased significantly during week-2WC and week-3WC compared to week-preWC and week-1WC; and ii) decreased significantly during week-4WC and week-postWC compared to week-2WC and week-3WC.

In the world (Table 1, Figure 6B), the mean value of effective R0 increased significantly during week-2WC compared to week-preWC. However, the mean value of effective R0 decreased significantly during i) week-postWC compared to week-preWC; ii) week-4WC and week-postWC compared to week-1WC; iii) week-3WC, week-4WC, and week-postWC compared to week-2WC); and iv) week-postWC compared to week-3WC.

## DISCUSSION

Our study investigated the potential consequences of the FIFA-WC 2022 for COVID-19 cases, deaths, and R0 in Qatar compared to the global situation. Specifically, we assessed the impact of a large-scale event conducted during the pandemic without implementing any COVID-19 restrictive measures, such as travel restrictions, social distancing, or mask-wearing, in Qatar.

We observed a significant increase in the number of COVID-19 confirmed cases in Qatar during week-3WC and week-4WC of the FIFA-WC 2022 compared to o week-preWC, week-1WC, and week-2WC. Intriguingly, there was a substantial decrease in the average number of cases during week-4WC and week-postWC compared to week-3WC. Furthermore, the increase in positive cases in Qatar was more pronounced than the global trend during the same period.

Mass gathering events have been linked to increased COVID-19 transmission among attendees [1, 2, 4, 5, 13–15]. For example, the Tokyo Olympic Games, held without spectators in July-August 2021, resulted in a surge of positive cases in Japan, prompting the Japanese government to declare an emergency situation during the Olympics. Despite efforts to minimise transmission risk, the highly transmissible Delta variant contributed to a spike in cases, hospitalisations, and deaths [4, 5].

The risk of COVID-19 transmission during large-scale events extends beyond the event venue itself, potentially impacting the home countries of attendees. This was demonstrated during the recent UEFA Euro 2020 tournament, where mass gatherings were followed by a significant increase in COVID-19 cases [13, 14]. For instance, in Finland, the rapid spread of the Delta variant was likely facilitated by travel-related imported cases from Euro 2020 [13, 14].

Interestingly, during the FIFA-WC, the global surge in positive cases followed a similar but less pronounced pattern to that of COVID-19 in Qatar. Mass gatherings occurred not only in Qatar but also worldwide, as families, friends, and sports enthusiasts convened to watch the event. The more pronounced effect in Qatar seems entirely expected due to the massive gathering of national and international fans, which may have facilitated the spread of COVID-19 among attendees in stadiums, fan zones, shopping centres, and other locations.

Our findings also revealed that by the end of the event (i.e. week-postWC), the number of positive cases had decreased, approaching its baseline values. This may be partially attributed to the potential benefits of Qatar’s healthcare system readiness during the FIFA-WC, which encompassed the presence of sufficient healthcare workers, the availability of medications, and dedicated COVID-19 hotlines to effectively mitigate the infection rate at mass gatherings [16, 17]. Moreover, Qatar had high vaccination coverage, which may have reduced the severity of infections and the overall number of cases, potentially mitigating the impact of mass gatherings on local COVID-19 transmission.

Qatar’s healthcare preparedness, including field hospitals and increased testing and tracing capacities, might also have contributed to early detection and isolation of cases, limiting large-scale outbreaks during the tournament. Furthermore, the study period coincided with the winter season in the Northern Hemisphere, which has been associated with increased respiratory infections, including COVID-19. It is possible that the global increase in cases and deaths during the study period was driven partly by seasonal factors [18], rather than the FIFA-WC itself.

Despite the absence of travel restrictions, social distancing, and mask-wearing measures during the FIFA-WC, other preventive measures, such as hand sanitizers at venues and frequent cleaning of high-touch surfaces, may have reduced the transmission risk. Analysing confirmed COVID-19 cases is essential for identifying the direct impact of the FIFA-WC on viral transmission and assessing the efficacy of public health measures in mitigating infection risk.

Furthermore, our analysis revealed a significant increase in the average number of COVID-19-related deaths in Qatar during week-2WC compared to week-preWC, week-3WC, week-4WC, and week-postWC. This suggests a temporary rise in COVID-19-related deaths during the event. However, it is challenging to establish a direct link between the organization of the event and the observed increase in deaths due to the long incubation period of the COVID-19 virus.

Examining the number of confirmed COVID-19-related deaths provides insight into the severity of the disease and the strain on healthcare systems during and after mass gathering events. An increase in deaths may indicate a higher disease burden and overwhelmed healthcare resources, which could have implications for future event planning and pandemic preparedness. Understanding the trend in confirmed deaths allows for a more comprehensive evaluation of the overall impact of such events on public health.

Our findings align with a study by Ahammer et al. [19], who investigated the impact of sports events on COVID-19 deaths in the US and the effectiveness of cancelling mass gatherings as a non-pharmaceutical intervention. The study reported that one additional National Basketball Association or National Hockey League game increased the cumulative number of COVID-19 deaths in urban areas and surrounding counties by 10.3%. It is essential to note that the study’s results are based on a time when facemasks were uncommon, vaccines were unavailable, and herd immunity had certainly not been reached.

During the Tokyo Olympic Games in Japan, a surge in COVID-19 cases and deaths was observed despite approximately 80% of the population being vaccinated [5]. Our findings suggest that the FIFA-WC in Qatar resulted in a significant number of deaths, but the number of cases was relatively small when compared to previous events such as the Tokyo Olympic Games. Moreover, the death rate in Qatar remained relatively low, below 0.05 per million population, compared to the global rate, ranging from 0.20 to 0.25. Nevertheless, the number of deaths returned to normal after the FIFA-WC. This observation might be attributable to the strength of herd immunity in Qatar compared to the global population, and Qatar’s predominantly young, expatriate population, which has an average age of 32.3 years, among other factors [20].

Additionally, our findings revealed that the mean R0 in Qatar increased significantly during week-2WC and week-3WC compared to week-preWC and week-1WC. However, it decreased significantly during week-4WC and week-postWC compared to week-2WC and week-3WC. For the global population, the mean R0 increased significantly during week-2WC compared to week-preWC. The mean R0 decreased significantly during week-postWC compared to week-preWC, week-4WC, and week-postWC compared to week-1WC, week-3WC, week-4WC, and week-postWC compared to week-2WC, and week-postWC compared to week-3WC. These results suggest that there was a notable increase in COVID-19 transmission during the FIFA-WC in Qatar and worldwide. However, the effective R0 began to decline towards the end of the event and afterward, indicating that the increase in transmission was temporary and likely associated with the mass gathering event. The R0, although increasing during the event, remained relatively low, between 0.8 and 1.5, which, along with the reduced fatality rate, was much lower than the R0 observed during other mass gathering events. For example, during the Tokyo Olympic Games, due to the Delta variant, the R0 was higher than 5 [11]. These low R0 rates before, during, and after the FIFA-WC offer valuable insights into the event’s low transmission dynamics of COVID-19 and the effectiveness of implemented public health measures in controlling disease spread globally, thanks to herd immunity.

In summary, several factors could have contributed to the observed trends in our study. First, the Qatari government’s efforts in implementing strict health and safety standards for the event, including the widespread availability of hand hygiene liquid, dedicated healthcare staff at mass gatherings, and health hotlines for any health issues, might have played a crucial role in mitigating the spread of COVID-19 during the FIFA-WC 2022. Second, Qatar’s highly vaccinated population [21], which reached over 90% coverage by the time of the event, might have provided a degree of herd immunity that limited the virus’s transmission.

### Study limitations

While our study provides valuable insights, it is important to recognize and address its inherent limitations. The first notable limitation pertains to the challenge of establishing causal inference. Given the observational nature of our study, we are unable to definitively establish a causal relationship between the FIFA-WC and the observed fluctuations in COVID-19 cases, fatalities, and R0 values in Qatar. The second limitation stems from the omission of key underlying confounding factors that could exert an influence on our results. Our investigation did not examine other potential contributing factors influencing COVID-19 metrics, such as shifts in public behaviour, vaccination rates, adherence to preventive measures, or unrelated infection control policies. These factors possess the potential to significantly impact the observed trends but remain unexplored in our study. A third limitation is due to discrepancies in data reporting, since our analysis fails to account for potential instances of underreporting, inconsistencies, or variations in data reporting methodologies across different countries. Such variations could potentially introduce bias and influence the precision of our comparative assessments. The fourth limitation revolves around the temporal scope of our study. The data collection window was relatively brief, spanning only a few weeks before, during, and after the FIFA-WC. This limited timeframe might not fully encapsulate the longer-term repercussions of the event on local and global COVID-19 caseloads. The effects of mass gatherings can unfold over the course of weeks or even months as individuals who were exposed return home, potentially triggering fresh outbreaks. In order to comprehensively comprehend the holistic impact of the FIFA-WC on COVID-19 transmission, it is imperative to undertake longitudinal investigations that analyse the prolonged effects on both local and global public health. The fifth limitation involves the extent to which our findings can be generalized. The applicability of our conclusions to other countries or events may be constrained due to the interplay of contextual variables that influence the impact of large-scale events on COVID-19 transmission dynamics. Notwithstanding these five limitations, our study provides valuable insights into the potential public health risks of conducting large events during an ongoing pandemic.

## CONCLUSIONS

The FIFA-WC 2022 in Qatar showed that large-scale sporting events can be safely organized during a pandemic with proper measures. Our study highlights the impact of the event on COVID-19 cases and deaths in Qatar. Despite initial challenges, effective infection control measures and local herd immunity helped control the virus’s spread. The study emphasizes the importance of high vaccination coverage, continuous monitoring, and collaboration between organizers, healthcare authorities, and governments. The FIFA-WC 2022 serves as a model for future events, emphasizing evidence-based decision-making and public health preparedness. Further research is needed to understand long-term effects and to refine best practices for event organization during pandemics.

## Data Availability

The data that support the findings of this study are openly available upon request from the corresponding author.
